# Cost-effectiveness of adding empagliflozin to the standard of care for patients with heart failure with reduced ejection fraction from the perspective of healthcare system in Malaysia

**DOI:** 10.3389/fphar.2023.1195124

**Published:** 2023-06-05

**Authors:** Siew Chin Ong, Joo Zheng Low, Stephan Linden

**Affiliations:** ^1^ Discipline of Social and Administrative Pharmacy, School of Pharmaceutical Sciences, Universiti Sains Malaysia, Pulau Pinang, Penang, Malaysia; ^2^ Hospital Sultan Ismail Petra, Ministry of Health, Kuala Krai, Malaysia; ^3^ Boehringer Ingelheim International GmbH, Ingelheim am Rhein, Germany

**Keywords:** empagliflozin, heart failure, cost-effectiveness, cost-utility, economic evaluation

## Abstract

**Objective:** The aim of this study was to determine the cost-effectiveness of adding empagliflozin to the standard of care *versus* SoC alone for the treatment of patients with heart failure (HF) with reduced ejection fraction (HF*r*EF) from the perspective of the Ministry of Health of Malaysia.

**Methods:** A cohort-based transition-state model, with health states defined as Kansas City Cardiomyopathy Questionnaire Clinical Summary Score (KCCQ-CSS) quartiles and death, was used to determine the lifetime direct medical costs and quality-adjusted life years (QALYs) for both treatment groups. The risks of all-cause death, cardiovascular death, and health state utilities were estimated from the EMPEROR-Reduced trial. The incremental cost-effectiveness ratio (ICER) was assessed against the cost-effectiveness threshold (CET) as defined by the country’s gross domestic product *per capita* (RM 47,439 per QALY) to determine cost-effectiveness. Sensitivity analyses were conducted to assess the key model parameters’ uncertainty in respect to the incremental cost-effectiveness ratio. A scenario analysis was performed using health states as defined by the New York Heart Association classes.

**Results:** Compared to SoC alone, empagliflozin + SoC for the treatment of HF*r*EF was more expensive (RM 25,333 vs. RM 21,675) but gained more health utilities (3.64 vs. 3.46), resulting in an ICER of RM 20,400 per QALY in the KCCQ-CSS model. A NYHA-based scenario analysis generated an ICER of RM 36,682 per QALY. A deterministic sensitivity analysis confirmed the robustness of the model in identifying the empagliflozin cost as the main driver of cost-effectiveness. The ICER was reduced to RM 6,621 when the government medication purchasing prices were used. A probabilistic sensitivity analysis with a CET of 1xGDP *per capita* reached 72.9% probability for empagliflozin + SoC against SoC being cost-effective.

**Conclusion:** Empagliflozin + SoC compared to SoC alone for the treatment of HF*r*EF patients was cost-effective from the perspective of the MoH of Malaysia.

## 1 Introduction

Heart failure (HF) is the terminal form of various cardiovascular (CV) disorders (i.e., acute coronary syndrome, cardio-rhythm disorder, valvular diseases, hypertension, and congenital heart diseases). The estimated worldwide prevalence of HF was about 60 million cases, with 50% of them having severe HF defined as symptomatic HF at rest (Lippi and Sanchis-Gomar, 2020) or reduced ejection fraction (Virani et al., 2020). HF is associated with frequent worsening of symptoms, thus substantially reducing quality of life and eventually leading to frequent hospitalisation and death (Savarese and Lund, 2017). The HF registry of Malaysia reported that the 30-day risk of all-cause readmission was 13% and went up to 45% within 1 year (National Heart Association Malaysia, 2021). After a worsening HF event, patients are more likely to have another episode of readmission than stable patients (Butler et al., 2020). The first prospective multinational Asian registry of patients with symptomatic HF (stage C), the ASIAN-HF Registry, reported that the overall mortality of patients with HF in the Southeast Asia region is 13.6% (MacDonald et al., 2020), which is comparable to developed countries (13%–18%) (Maggioni et al., 2013; Virani et al., 2020). In Malaysia, the estimated total cost of HF in 2012 was RM 785 million (National Heart Association of Malaysia, 2019). Inpatient cost is the main cost driver of the healthcare cost of HF in Malaysia and accounts for about 90% of the total healthcare costs of HF (Shafie et al., 2019).

The primary goals of treatment in HF patients are to reduce hospitalisation due to heart failure (hHF), CV death, and to improve symptoms and health-related quality of life (HRQoL) (McDonagh et al., 2021). Evidence-based therapy can avert CV death and hHF in HF patients with reduced ejection fraction (HF*r*EF) (Yancy et al., 2017; McDonagh et al., 2021), which accounts for two-thirds of patients with this syndrome (National Heart Association Malaysia, 2021). Currently, the available treatments for patients with HF*r*EF are pharmacological therapy and device implantation in selected patients only. Optimal pharmacological therapy includes renin–angiotensin–aldosterone system inhibitors, beta-blockers, mineralocorticoid inhibitors (MRA), and sodium–glucose cotransporter type 2 inhibitors (SGLT2i) (Yancy et al., 2017; McDonagh et al., 2021). Empagliflozin is a medication from SGLT2i that reduces the risk of primary composite outcomes of CV death or hHF by 25% when compared to the standard of care (SoC) (Packer et al., 2020). The clinical benefit of empagliflozin on the primary composite outcome is driven by the reduction in the risk of hHF by 30% among HF*r*EF patients receiving empagliflozin.

Empagliflozin is employed as a cost-effective treatment in patients with HF*r*EF in Taiwan, Japan, South Korea, Australia, Singapore (Liao et al., 2021), and China (Jiang et al., 2021). However, the cost-effectiveness of empagliflozin in addition to SoC for treating HF*r*EF patients is not readily available in Malaysia. This analysis is essential for decision-makers to justify allocating scarce resources to adopting empagliflozin as part of the treatment regimen for HF*r*EF. Therefore, the study’s objective is to determine the cost-effectiveness of adding empagliflozin to SoC in patients with HF*r*EF from the perspective of the Ministry of Health (MoH) of Malaysia.

## 2 Methods

### 2.1 Model description

**FIGURE 1:**
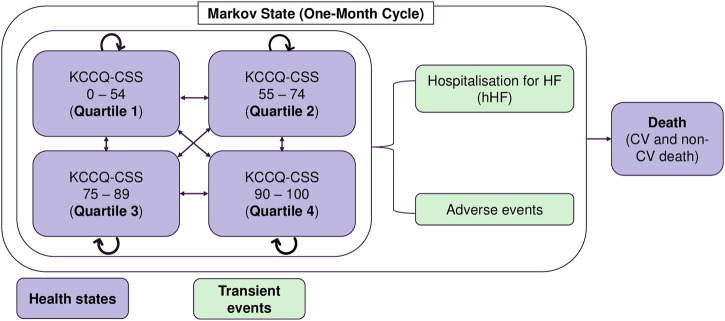
Markov model structure. AE: adverse events; CSS: clinical summary score; CV: cardiovascular; hHF: hospitalisation due to heart failure; ICER: incremental cost-effectiveness ratio; KCCQ: Kansas City Cardiomyopathy Questionnaire; RM: Ringgit Malaysia.

A validated cohort-based transition-state Markov model was adopted from the cost-effectiveness model (CEM) of empagliflozin + SoC vs SoC monotherapy submitted to the National Institute for Health and Care Excellence (NICE) (National Institute for Health and Care Excellence, 2022). The CEM was conducted from the perspective of the Malaysian Ministry of Health as the payer for the Malaysian healthcare system. The Markov model simulated the clinical course of HF*r*EF patients through health states based on the Kansas City Cardiomyopathy Questionnaire Clinical Summary Score (KCCQ-CSS) quartiles (Figure 1). As the KCCQ-CSS health states are a patient-centric approach that is better aligned with clinical symptoms and survival, it is preferred over the New York Heart Association (NYHA) classification (McEwan et al., 2020; National Institute for Health and Car e Excellence, 2021). The progression of HF*r*EF was simulated using five health states with health state-specific values: KCCQ-CSS quartile 1 (0–54), KCCQ-CSS quartile 2 (55–74), KCCQ-CSS quartile 3 (75–89), KCCQ-CSS quartile 4 (90–100), and death. The patient cohort in CEM was based on the baseline KCCQ-CSS quartiles distribution in the EMPEROR-Reduced trial. Patients could either transition to a higher quartile (less disease burden) or a lower quartile (more disease burden), remain in the same health state, or die. The model used a lifetime horizon with a monthly cycle length and half-cycle correction to account for the chronic and progressive nature of the disease, consistent with previous HF economic models (National Institute for Health and Care Excellence, 2012; National Institute for Health and Care Excellence, 2016; Di Tanna et al., 2019).

The model captured the incidence of hHF and adverse effects related to treatment as transient events. Patients in each KCCQ-CSS quartile experienced a monthly risk of hHF, CV death, or non-CV death. The transition probability matrix for transitions between the different KCCQ-CSS quartiles was applied to the remaining number of patients in the ‘alive’ health states to calculate the health state distribution in the next cycle. Patients could discontinue treatment with empagliflozin in each cycle. After discontinuation, patients received SoC treatment until death or the end of the modelled time horizon. Patients who discontinued treatment with empagliflozin experienced the same event rates and health state transition probabilities as those receiving SoC. Multiple admissions for HF were permitted over the entire model’s time horizon for a more realistic representation of the clinical journey. The adverse events modelled were urinary tract infection, genital mycotic infection, acute renal injury, hepatic injury, hypotension, hypoglycaemic event, and bone fracture.

Only direct medical costs were included in the model. These were associated with medication acquisition, CV death, hHF, disease management, and treating adverse effects. Utilities were accrued based on the duration spent in each KCCQ-CSS quartile, adjusted for disutilities associated with hHF and adverse events.

A 3% discount rate was applied to future costs and outcomes (Pharmaceutical Services Programme, 2019). The Markov model development and analyses were performed using Microsoft Excel^®^ (Microsoft, USA). Ethical approval for this study was obtained from the Medical Research and Ethics Committee (MREC), Ministry of Health of Malaysia (NMRR ID-21-02,128-9 KR).

### 2.2 Cohort population

The modelled patient population was the intention-to-treat (ITT) population from the EMPEROR-Reduced trial, which corresponded to the anticipated licensed indication of empagliflozin for the treatment of HF*r*EF. The initial distribution of patients according to the KCCQ-CSS quartiles was as follows: KCCQ-CSS quartile 1 (24.3%), KCCQ-CSS quartile 2 (25.1%), KCCQ-CSS quartile 3 (27.2%), and KCCQ-CSS quartile 4 (23.4%) (Supplementary Appendix A Table A1). Adult participants (aged ≥18 years) who had been diagnosed with HF with left ventricular ejection fraction (LVEF) ≤ 40% and NYHA functional classes II-IV were simulated in CEM. The majority of the patients were male (76%), and about three-quarters (75.1%) of the modelled patients were classified as NYHA class II (Supplementary Appendix A Table A1). Patients with HF originating from an ischaemic aetiology were 51.7%. HF*r*EF patients with type 2 diabetes mellitus (T2DM) were about half (49.8%) of the total population. The mean [standard deviation (SD)] starting age of the cohort was 60 (13.6) years, which was the mean age of HF patients reported in the Malaysian Heart Failure Registry (MyHF) (Abidin et al., 2021). The details of the inclusion criteria and demographic characteristics of the trial patients are provided in the published article (Packer et al., 2020).

### 2.3 Intervention and comparator

The intervention was empagliflozin at a dose of 10 mg once daily in addition to the SoC for the HF*r*EF patients. The comparator of this study was SoC only. The SoC comprised renin–angiotensin–aldosterone system blockers (either angiotensin receptor–neprilysin inhibitor [ARNi], angiotensin-converting enzyme inhibitor [ACEi], or angiotensin receptor blocker [ARB]), beta-blockers, MRA, ivabradine, diuretics, and cardiac devices when indicated. The utilisation patterns were estimated based on the EMPEROR-Reduced trial. Each medication in the SoC was assumed to have the same utilisation rate across all KCCQ-CSS quartiles. In the model, empagliflozin acted to delay disease progression and reduce the incidence of hHF and CV death in patients with HF*r*EF. The treatment effect of empagliflozin was assumed to be unaffected by different combinations of background therapies. This assumption was supported by the findings of the EMPEROR-Reduced trial *post hoc* analysis, which found that the clinical benefits of empagliflozin were independent of background therapies (Packer et al., 2020).

### 2.4 Input parameters

#### 2.4.1 Clinical data

The model also considered improvement (ascent) or progression (descent) of disease *via* the transition of patients between KCCQ-CSS quartiles. Separate transition probabilities for movement between different KCCQ-CSS health states were obtained from the EMPEROR-Reduced trial for each treatment group. There were three transition probability matrices: baseline to week 12 (months 1–3), week 12 to week 32 (months 4–8), and week 32 to week 52 (months 9+). These reflected the different inflexion points observed in the data (Supplementary Appendix A Table A2).

Data obtained from the EMPEROR-Reduced trial were statistically analysed to derive the rates and risk of events and the efficacy of empagliflozin. The EMPEROR-Reduced trial was selected as the primary source for the clinical benefits of empagliflozin because the trial was a multinational phase III trial that investigated the efficacy of empagliflozin and SoC against SoC alone on the composite outcomes of hHF or CV death in approximately 3,370 HF patients with LVEF<40%. In addition, the management of HF with medical therapies and devices is consistent with local and international clinical practice guidelines; thus, it is highly relevant to the actual clinical practice in the hospital setting. Statistical analyses of data from the EMPEROR-Reduced trial produced separate equations for all-cause death, CV death, hHF, and treatment discontinuation with the KCCQ-CSS quartile as the time-varying predictor (Supplementary Appendix A Table A3). Furthermore, the treatment effects of empagliflozin were included as a covariate in the hHF, death (CV and all-cause), and treatment discontinuation equations.

The risk equation for hHF events was derived using the ITT population of the EMPEROR-Reduced trial. The rate of first and recurrent hHF was estimated from a model constructed using a Poisson model fitted to patient-level data with generalised estimating equations to account for the repeated measures on patients. The model included treatment and time-varying KCCQ-CSS quartiles as predictors.

A parametric survival analysis using a Weibull distribution allowed for extrapolation of time to all-cause death and CV-related death as a function of treatment. KCCQ-CSS quartiles health states were used to estimate disease progression beyond the EMPEROR-Reduced trial duration. The Akaike information criterion values for various distributions were compared to determine the best distribution fitted to the equations (with lower values indicating better fit)—in this case, Weibull. This distribution enables a more accurate estimation of the survival benefit of empagliflozin beyond the duration of the clinical trial. An alternative distribution (exponential) was considered in the sensitivity analysis. The clinical events in a clinical trial, such as time-to-events (e.g., mortality), are often censored; therefore, not all the events of interest will be noticed for all the participants at the end of a trial. Generally, data censoring occurs because some participants did not experience the event of interest when the trial ended or were lost to follow-up. Data extrapolation for time to events will better estimate the efficacy of a new intervention beyond the trial period (Latimer, 2013). Deaths attributable to non-CV causes during each model cycle were calculated based on the difference between the all-cause death and CV death rates, which were estimated using the parametric equations or the difference between the rates of age- and sex-specific all-cause death and CV death for the general Malaysian population, whichever was the highest. This adjustment ensured that the non-CV death in the CEM was at least as high as it was in the general Malaysian population.

Parametric survival analysis was applied to estimate the time to empagliflozin treatment discontinuation (using exponential distribution). The alternative distribution (Weibull) was observed in the EMPEROR-Reduced trial. The treatment and time-varying KCCQ-CSS quartiles were considered predictors for empagliflozin discontinuation in the analysis. After discontinuing empagliflozin + SoC, patients were assumed to receive SoC and thus experience the same risk of clinical events, costs, and utility decrements as patients on SoC.

The CEM included the probability of experiencing adverse events from the treatment of HF and was modelled assuming a constant incidence rate. The model included the adverse events associated with empagliflozin with SoC and SoC monotherapy, and their respective rates were obtained from the EMPEROR-Reduced trial (Supplementary Appendix A Table A4).

#### 2.4.2 Cost

This study adopted the perspective of the Malaysian healthcare system, whereby only direct medical costs were included. All costs are presented in 2021 Malaysian Ringgits (RM).

Drug costs for empagliflozin 10 mg and SoC therapies were obtained from the IQVIA dataset that had the market sale prices for Malaysia. The indicated strength and dosage for each drug were based on the Ministry of Health (MoH) Medication Formulary, which includes information on medications licensed to be used in MoH facilities. The monthly acquisition costs of SoC were based on the recommended doses of each active ingredient as confirmed by the cardiologists from the MoH facilities. The weighted average costs of each active ingredient were calculated using the percentage of utilisation (Supplementary Appendix A Table A5) from the in-house local study (Ong et al., 2022). Then, the weighted average cost of SoC was computed using the utilisation rate of HF medication classes reported in the EMPEROR-Reduced trial (at baseline) and the weighted average monthly costs of each class. Finally, the cost associated with each treatment regimen (i.e., empagliflozin + SoC and SoC monotherapy) in the model was computed (Supplementary Appendix A Table A6). The drug acquisition costs for empagliflozin + SoC and SoC monotherapy were RM 285.83 and RM 175.17, respectively.

The cost of hHF per admission was obtained from the in-house local study (Ong et al., 2022) (Supplementary Appendix A Table A7). The cost of hHF consisted of hospitalisation care, medication, diagnostic tests, and procedures. The cost of CV death was estimated from the cost analysis of the management of T2DM in the Action in Diabetes and Vascular Disease (ADVANCE) study using only the data for Malaysia (Clarke et al., 2010). The cost of CV death was defined as the cost of fatal events due to major coronary, cerebrovascular, and HF; it was first derived separately for male and female people. The weighted cost of each fatal event was weighted by the proportion of male and female people among the HF population obtained from the Malaysia Heart Failure Registry (National Heart Association Malaysia, 2021). The average cost of CV death was then weighted by the number of patients who died due to each fatal event in 2020. Appendix B provides additional details on how the cost of CV death was derived. The non-CV death cost was assumed to be the same between the comparator and intervention groups, thus incurring no additional cost for the MoH of Malaysia.

The HF-related disease management costs and frequencies associated with HF clinic follow-up visits were obtained from the in-house local study (Ong et al., 2022). The resources (frequency of visits and cost) utilised by patients with HF*r*EF were converted from an annual to monthly frequency and assumed to be the same for all KCCQ-CSS quartiles. Then, the disease management costs for all KCCQ-CSS quartiles were computed based on the frequency of visits and unit cost.

The costs of managing adverse events were calculated as weighted average costs based on the proportion of the type of care received (inpatient vs. outpatient visit), as estimated by the cardiologists from the MoH facilities, and the unit cost of each visit (Supplementary Appendix A Table A8). The cost of inpatient care was obtained from the Malaysia Disease-Related Group (DRG) case-mix database. The cost of outpatient care was derived from the resource utilisation during outpatient visits as determined by experts’ opinions on the treatment algorithm.

The costs were adjusted using the consumer price index (CPI) health domain to the 2021 Malaysian Ringgit value (Department of Statistics Malaysia, 2022).

#### 2.4.3 Utility

The utility values were used to evaluate the impact of health states and clinical events on the HRQoL. The quality-adjusted life years (QALYs) accrued for each cycle were determined by subtracting utility reductions attributable to hHF and adverse events from the health state utilities.

Due to the unavailability of utility data for Malaysian HF patients, utility values associated with KCCQ-CSS quartiles and disutility values associated with adverse events and hHF were obtained from the ITT population pooled analysis in the EMPEROR-Reduced trial. The EQ-5D-5L questionnaire responses of patients were mapped to EQ-5D-3L (Alava et al., 2017) scores and converted into utility index scores using the appropriate value sets for the United Kingdom (Dolan, 1997). A linear mixed regression model was fitted to account for repeated utility measurement on the same patients, baseline demographic characteristics, comorbidities, health states, and clinical events (Supplementary Appendix A Table A9).

The impact of hHF and adverse events on HRQoL was captured as a one-off decrement in the proportion of the cohort who had experienced the events in each cycle. Decrements associated with the clinical event (hHF) and adverse events were applied over a duration equal to that of the model cycle length. The disutility values associated with urinary tract infection, genital mycotic infection, acute renal failure, and hypotension were obtained from Sullivan et al (Sullivan and Ghushchyan, 2006; Sullivan and Ghushchyan, 2016). Disutility values for other adverse events (hepatic injury, volume depletion, and bone fracture) were generated from a patient-level analysis of the EMPEROR-Reduced trial because the trial values were deemed to be more reflective of the population of interest compared to the values reported in the literature. The disutility value for hypoglycaemic events was obtained from the CEM of empagliflozin previously submitted to NICE (National Institute for Health and Care Excellence, 2011).

### 2.5 Outcome measures

The primary outcomes of this study were the total cost, total QALYs, incremental cost, incremental QALYs, and incremental cost-effectiveness ratio (ICER). The secondary outcomes of the CEM were the number of hHF, CV death, non-CV death, life years gained, and incremental cost per life year gained. The ICER was defined as the ratio of the difference in the total healthcare cost between the two treatment groups to the healthcare outcomes; it was expressed as cost per life year gained or cost per QALY gained. The ICER was compared to the cost-effectiveness threshold (CET) to determine the cost-effectiveness of empagliflozin. Adding empagliflozin to SoC in HF patients was deemed to be cost-effective when the ICER generated from this study was below the CET. The CET based on one-time GDP *per capita* in 2021was RM 47,439 per QALY (The World Bank, 2022).

### 2.6 Sensitivity analyses

Deterministic sensitivity analysis (DSA) was performed to determine the impacts of varying model inputs within the plausible range on the ICER and to identify model drivers. A tornado diagram displayed the results of the one-way sensitivity analyses.

A multivariate probabilistic sensitivity analysis (PSA) was conducted to examine how the ICER was affected by simultaneous variations in different model inputs within their feasible ranges based on the assumed probability distributions. The parameters included in the PSA are summarised in Supplementary Appendix A Table A10. The simulation was repeated 1,000 times to generate a range of ICERs for a given set of model inputs. The generated ICERs were then summarised and plotted on a cost-effectiveness plane. Finally, a cost-effectiveness acceptability curve was plotted to illustrate the cost-effectiveness probability of the addition of empagliflozin to the SoC at a given CET.

### 2.7 Scenario analyses

Scenario analyses were performed to evaluate certain scenarios that significantly impacted the ICER. A scenario analysis was conducted using the NYHA functional class as the health state instead of the KCCQ-CSS quartiles. The NYHA functional classification is commonly used in routine clinical practice to classify HF patients according to the severity of clinical symptoms and physical functionality. The CEM included four health states in this scenario: NYHA I, NYHA II, NYHA III/IV, and death. NYHA class III and class IV were combined due to the low number of patients in class IV. Similarly to the CEM model using KCCQ-CSS quartiles, HF patients could transfer between different health states and were subjected to the health state probability of experiencing hHF and death (CV and non-CV) during each cycle. Appendix C reports the transition matrices, risk equation for all-cause death, CV death, hHF, treatment discontinuation, utility, and disease management costs for the NYHA-based model. In accordance with the EMPERIOR-Reduced trial, the starting age of the cohort was increased from 60 years in the base case to 67 years. In addition, a different time horizon was explored to determine the uncertainties caused by the duration of treatment. Lastly, the effects of using the medication acquisition costs of MoH-funded hospitals on the ICER were also explored. The costs were obtained from the average acquisition costs by the procurement unit of a fully funded MoH hospital.

All patient subgroups in the EMPEROR-Reduced trial benefited from treatment with empagliflozin + SoC compared to SoC alone. The reduction in the risk primary composite outcome (hHF or CV death) was shown to be consistent across multiple subgroups, including baseline T2DM status, age (<65 years or ≥65 years), sex race, baseline body mass index, and prior ARNi used (Packer et al., 2020). Thus, only the ITT population was considered in the CEM.

## 3 Results

### 3.1 Base-case cost-effectiveness analysis result

Table 1 displays the discounted results of the base-case analysis of adding empagliflozin to SoC against SoC alone for treating HF*r*EF over a lifetime horizon. SoC monotherapy was associated with 4.84 life years, 3.46 QALYs, and a total cost of RM 21,675 per patient. Adding empagliflozin to SoC increased the accrued life years and QALYs by 0.14 and 0.18 per person, respectively, but at an additional cost of RM 3,658 per person. Treatment of HF*r*EF patients with empagliflozin as an add-on to SoC was cost-effective against SoC monotherapy, with an ICER of RM 20,400 per QALY. The ICER generated from the CEM was well below the CET of 1xGDP *per capita* (RM 47,439/QALY). Essentially, the treatment with empagliflozin + SoC against SoC alone was cost-effective.

**TABLE 1:** Base-case results for the cost-effectiveness of adding empagliflozin to the standard of care.

Outcome	Empagliflozin + SoC	SoC	Incremental
Total Cost (RM)	25,333	21,675	3,658
Total LYs	4.98	4.84	0.14
Total QALYs	3.64	3.46	0.18
ICER, Cost per LY gained (RM/LY)	26,268		
ICER, Cost per QALY gained (RM/QALY)	20,400		

ICER: incremental cost-effectiveness ratio; LY: life years; QALYs: quality-adjusted life years; RM: ringgit malaysia; SoC: standard of care.

Treatment with empagliflozin + SoC was associated with a reduction in hHF and CV death incidence by 19.3% and 5.8% compared to SoC (Table 2). In addition, HF patients treated with empagliflozin + SoC had higher QALYs (+0.18) compared to SoC monotherapy. The incremental QALYs gained were driven by the increased life years and longer time spent in the alive state, particularly among patients in KCCQ-CSS quartile 4 (+0.20 life years and +0.17 QALYs). Furthermore, the incidence of hHF averted by the treatment of empagliflozin + SoC contributed to 0.04 QALYs gained. Treatment with empagliflozin + SoC was also associated with a lower risk of developing acute renal failure, hepatic injury, and hypoglycaemic events compared to SoC monotherapy. Conversely, treatment with empagliflozin + SoC led to a higher rate of adverse events such as urinary tract infection, genital mycotic infection, volume depletion, hypotension, and bone fracture.

**TABLE 2:** Summary of clinical and cost outcomes for the base-case scenario.

Clinical outcomes	Empagliflozin + SoC	SoC	Incremental
Event rates (per 100 patient years)			
HF hospitalisation	17.02	21.10	−4.08
CV death	9.58	10.17	−0.59
Non-CV death	8.06	8.03	0.04
Adverse events			
Urinary tract infection	3.99	3.76	0.23
Genital mycotic infection	1.06	0.53	0.53
Acute renal failure	8.46	9.02	−0.56
Hepatic injury	3.58	3.83	−0.25
Volume depletion	9.07	8.76	0.31
Hypotension	8.02	7.69	0.33
Hypoglycaemic event	1.22	1.25	−0.03
Bone fracture	1.97	1.89	0.08
Time on treatment (undiscounted), LYs, and QALYs (discounted) per patient			
Time receiving empagliflozin (years)	3.55	N/A	
**Total LYs**	**4.98**	**4.84**	**0.14**
KCCQ-CSS Quartile 1	0.77	0.84	−0.07
KCCQ-CSS Quartile 2	1.02	0.99	0.03
KCCQ-CSS Quartile 3	1.32	1.34	−0.02
KCCQ-CSS Quartile 4	1.88	1.67	0.20
**Total QALYs**	**3.64**	**3.46**	**0.18**
KCCQ-CSS Quartile 1	0.46	0.51	−0.04
KCCQ-CSS Quartile 2	0.73	0.71	0.02
KCCQ-CSS Quartile 3	1.04	1.06	−0.02
KCCQ-CSS Quartile 4	1.61	1.44	0.17
Loss due to hHF	−0.207	−0.252	0.04
Loss due to AEs	−0.005	−0.005	0.00

**Cost Outcomes**	**Empagliflozin + SoC**	**SoC**	**Incremental**
Cost outcomes (discounted), per patient			
**Drug acquisition cost (RM)**	**14,753**	**10,168**	**4,585**
**Clinical event management cost (RM)**	**5,600**	**6,596**	**−996**
HF hospitalisation	4,414	5,369	−955
CV death	1,186	1,227	−41
**AE management cost (RM)**	**3,291**	**3,269**	**22**
Urinary tract infection	34	31	3
Genital mycotic infection	17	8	9
Acute renal failure	1,331	1,382	−51
Hepatic injury	571	595	−24
Volume depletion	443	416	28
Hypotension	510	475	36
Hypoglycaemic event	42	42	0
Bone fracture	343	320	23
**Disease management cost (RM)**	**1,689**	**1,642**	**47**
KCCQ-CSS Quartile 1	260	285	−24
KCCQ-CSS Quartile 2	345	334	11
KCCQ-CSS Quartile 3	446	455	−8
KCCQ-CSS Quartile 4	637	568	69
**Total cost (RM)**	**25,333**	**21,675**	**3,658**

AE: adverse events; CSS: clinical summary score; CV: cardiovascular; HF: heart failure; hHF: hospitalisation due to heart failure; LY: life year; KCCQ: kansas city cardiomyopathy questionnaire; QALY: quality-adjusted life year; RM: ringgit malaysia; SoC: standard of care.

The main cost driver of incremental costs associated with empagliflozin + SoC treatment was the drug acquisition cost of empagliflozin. This was partially offset by cost savings from the reduction in the incidence of CV death and hHF relative to SoC. The costs of adverse event management were nearly equivalent for both arms. Treatment with empagliflozin + SoC increased the incidence of volume depletion and hypotension. However, the increased costs associated with these adverse events were partially offset by the decreased incidence of acute renal failure and hepatic injury, which are more expensive to treat than in the other modelled adverse events. The life years gained from the treatment of empagliflozin + SoC translated to a higher disease management cost in the intervention arm. This is because the disease management cost was calculated based on the survival duration. Furthermore, patients treated with the intervention arm remained in quartile 4 for a longer duration, leading to the highest incremental disease management cost in this health state.

### 3.2 Deterministic sensitivity analysis

**FIGURE 2:**
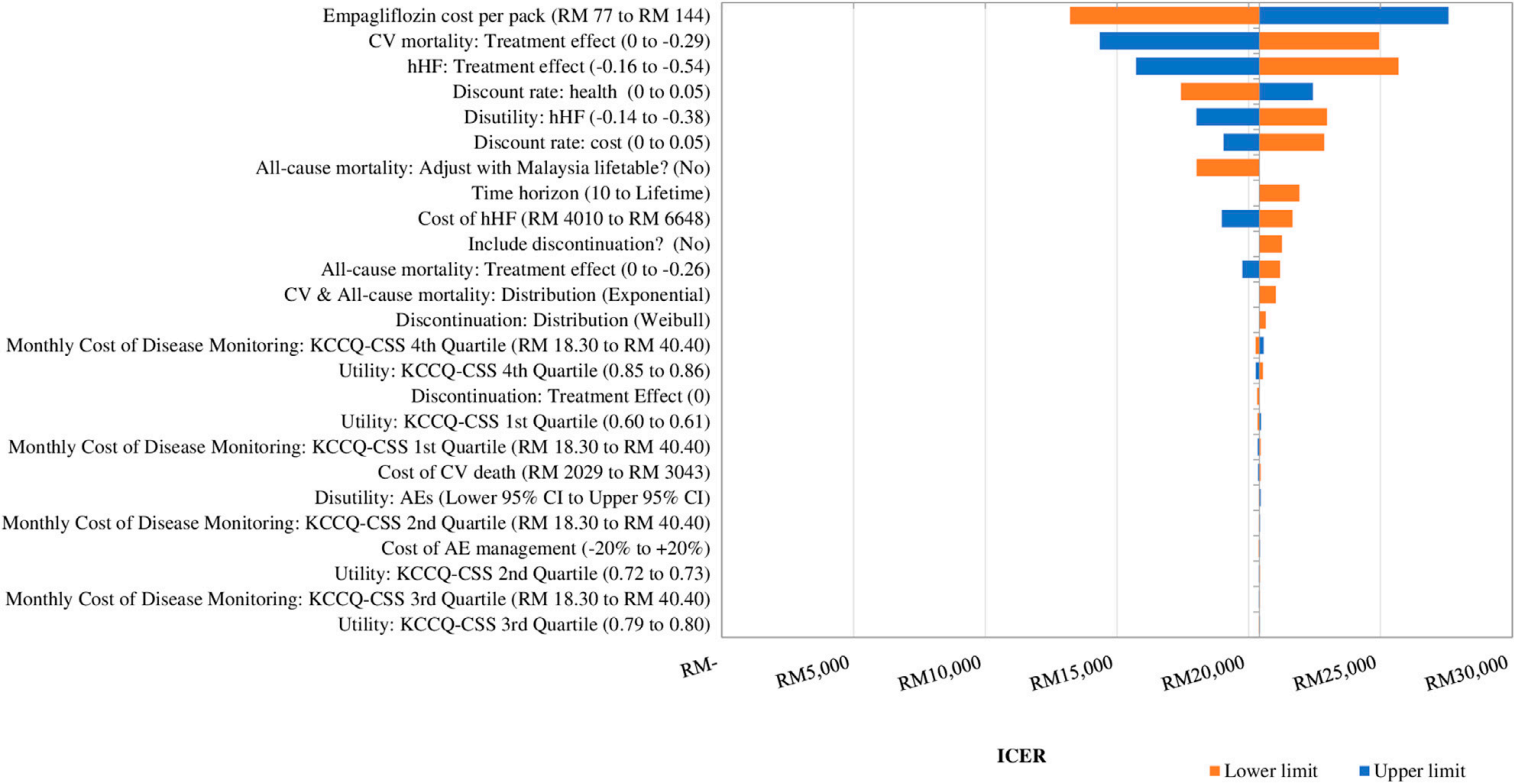
Tornado diagram showing the deterministic sensitivity analysis of the cost-effectiveness model simulation. GDP: gross domestic product; QALY: quality-adjusted life year; RM: Ringgit Malaysia; SoC: standard of care. 1xGDP *per capita* is RM 47,439 per QALY.

Figure 2 displays the DSA results. The drug acquisition cost of empagliflozin was the main driver of the cost-effectiveness of adding empagliflozin to SoC against SoC alone. The ICER changed by 35.2% when the acquisition cost of empagliflozin changed by 30%. The second most impactful parameter was the treatment effect of empagliflozin + SoC in preventing CV death. The ICER increased by 22.3% to RM 24,949 per QALY when the effect of empagliflozin + SoC associated with CV death was assumed to be the same as SoC alone, but the ICER dropped to RM 14,346 per QALY at the upper value (Supplementary Appendix D Table D1). The other drivers of the CEM were the treatment effect associated with hHF, the discount rate on health, and disutility for a hHF event.

### 3.3 Probabilistic sensitivity analysis

**FIGURE 3:**
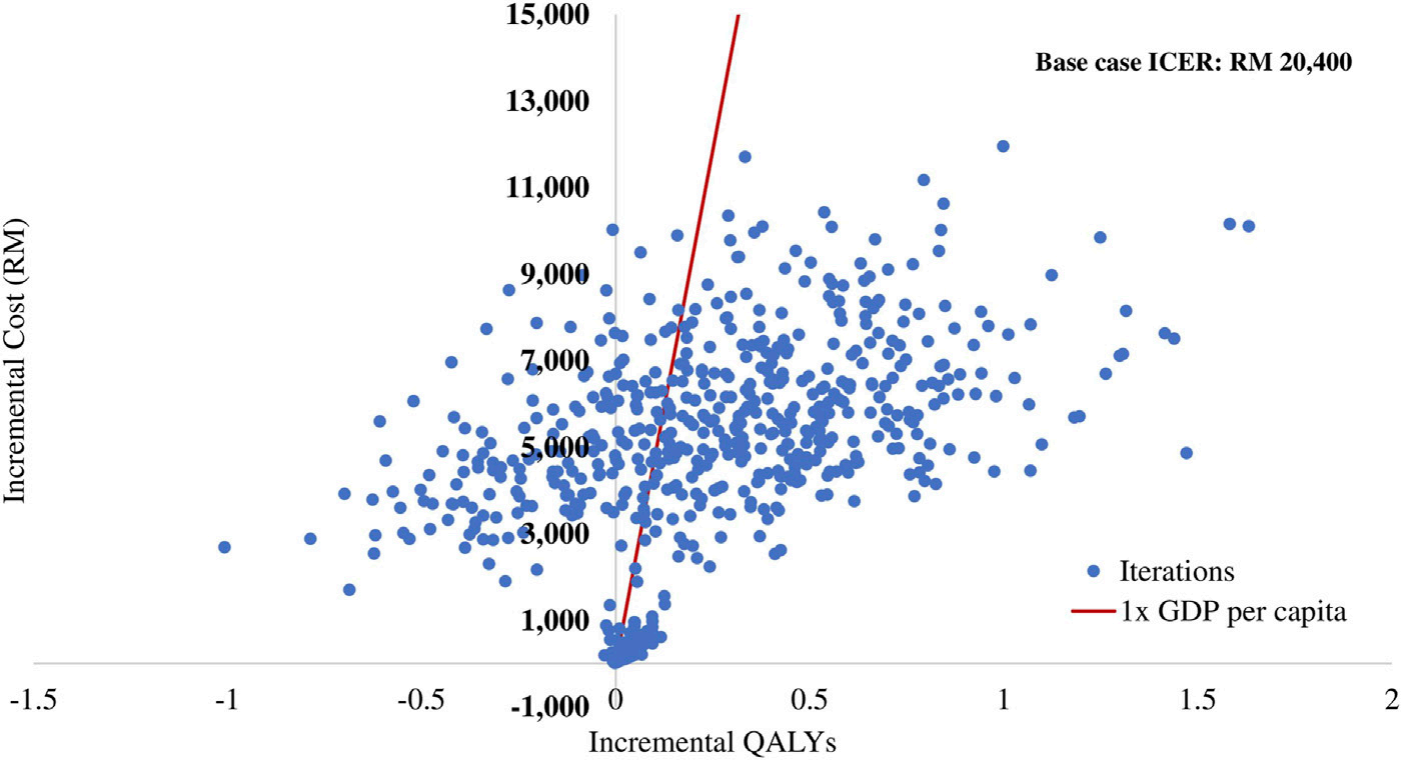
Scatter plot of incremental cost and incremental quality-adjusted life year for empagliflozin + SoC vs SoC. QALY: quality-adjusted life year; RM: Ringgit Malaysia; SoC: standard of care.

**FIGURE 4:**
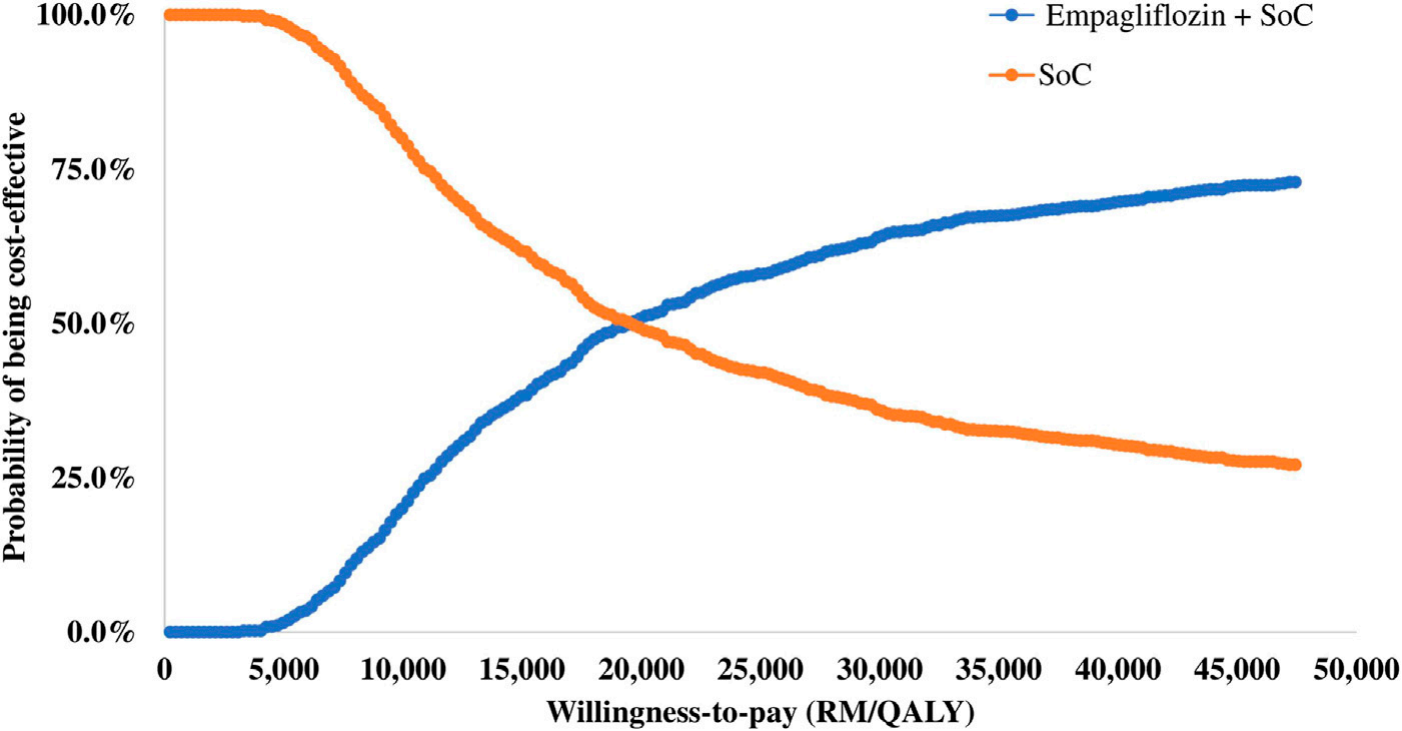
Cost-effectiveness acceptability curve for empagliflozin + SoC vs SoC.

The results of the PSA are summarised in the cost-effectiveness plane (Figure 3) and the cost-effectiveness acceptability curve (Figure 4). Of 1,000 iterations, 82.8% were located in the north-east quadrant of the cost-effectiveness plane, thus indicating that adding empagliflozin to the SoC was more costly but more treatment effective than SoC alone. In addition, among these, 72.9% of the simulation’s replications produced an ICER value below the 1xGDP *per capita* (RM 47,439 per QALY). Furthermore, the average ICER from the PSA analysis was RM 20,266, similar to the ICER generated from the base-case scenario of RM 20,400 (Supplementary Appendix D Table D2). The small difference between the ICER derived from the base-case scenario and the PSA reflect the robustness of the CEM.

### 3.4 Scenario analysis

A scenario analysis was conducted wherein the health states were defined as NYHA functional classes instead of KCCQ-CSS quartiles. In the NYHA-based model, the total cost for empagliflozin + SoC was RM 23,970 per patient, which was RM 3,395 higher than the SoC alone over the lifetime horizon (Supplementary Appendix E Table E1). Similarly, the empagliflozin add-on to the SoC resulted in increased life years (+0.05) and QALYs (+0.09) against SoC alone. As a result, the deterministic analysis based on the NYHA model generated an ICER of RM 36,682 per QALY, which was below the CET.

The empagliflozin + SoC arm was associated with a reduction in hHF and CV death incidence rates but a slight increase in the non-CV death rate (Supplementary Appendix E Table E2). Furthermore, the clinical benefits of empagliflozin in reducing the incidence of hHF and CV death translated in cost saving; these partially offset the cost of acquiring empagliflozin in the intervention arm. However, the disease management cost of the intervention arm increased by RM 18 per patient due to the increased lifespans of HF*r*EF patients.

The ICER decreased from RM 34,365 per QALY to RM 21,922 per QALY when the time horizon was increased from 1 year to 10 years (Table 3). In addition, when the cohort’s starting age was 67 years (60 years for the based-case analysis), the ICER increased to RM 22,268 per QALY. When the MoH acquisition cost was applied, the ICER for KCCQ-CSS and NYHA health states decreased to RM 6,627 per QALY and RM 12,188 per QALY, respectively.

**TABLE 3:** Findings of scenario analyses presented as ICER.

Scenarios	Incremental cost (RM)	Incremental QALYs	ICER (RM/QALY)
**Base case**	3,658	0.18	20,400
**Time horizon**			
1 year	819	0.02	34,365
5 years	2,679	0.10	25,903
10 years	3,440	0.16	21,922
**Source of medication cost**			
**IQVIA**			
NYHA	3,395	0.09	36,682
**MoH acquisition cost**			
KCCQ-CSS	1,188	0.18	6,627
NYHA	1,128	0.09	12,188
**Cohort starting age**			
67 years	3,125	0.14	22,268

ICER: incremental cost-effectiveness ratio; QALY: quality-adjusted life year; RM: ringgit malaysia.

CSS: clinical summary score; CV: cardiovascular; KCCQ: kansas city cardiomyopathy score.

* Adverse events: urinary tract infection, genital mycotic infection, acute renal injury, hepatic injury, hypotension, hypoglycaemic event, and bone fracture.

## 4 Discussions

This study is the first cost-utility study that investigated the addition of empagliflozin to the SoC in the treatment of HF*r*EF in Malaysia. It was performed based on the clinical efficacies of empagliflozin derived from the EMPEROR-Reduced trial and localised cost data. The base-case analyses indicated that empagliflozin + SoC was cost-effective compared to SoC alone. The prevention of hHF primarily drove the cost-effectiveness of empagliflozin, resulting in more life years and QALYs gained among patients treated with empagliflozin. Moreover, the cost-savings from the prevention of hHF partially offset the acquisition cost of empagliflozin.

Cost-utility analyses of empagliflozin + SoC against SoC alone were conducted in Thailand (Krittayaphong and Permsuwan, 2022), Taiwan (Liao et al., 2021), the United Kingdom (National Institute for Health and Care Excellence, 2022), and China (Jiang et al., 2021; Lin et al., 2022; Sang et al., 2022; Tang and Sang, 2022). Although these studies reported that empagliflozin + SoC was more cost-effective than SoC alone, a localised country-based economic evaluation need to be conducted in Malaysia due to concerns about the applicability and generalisability of findings from other cost-utility analyses. The ICER generated from the studies performed in other countries studies cannot be applied to the local setting because of the differences in the i) resources and associated costs, ii) acquisition costs of medications, iii) healthcare system, iv) health utilities valuations, v) analysis perspective, and vi) discount rate.

Liao *et al.* performed a cost-effectiveness evaluation using a two health states model (stable HF and death) with hospitalisation as a transient event from the healthcare payer perspective of Taiwan (Liao et al., 2021). The study reported that the ICER generated for the add-on empagliflozin against SoC alone was USD 20,508 per QALY, below the country’s 1x GDP *per capita* of USD 25,000. Subsequently, the author also examined the cost-effectiveness of adding empagliflozin to the SoC for different countries from the Southeast Asia–Pacific region using localised cost data. The ICERs generated in the study were consistently below these countries’ 1xGDP *per capita* WTP threshold, except for Thailand. Using the two-states model to simulate the analysis in the CEM posed a few concerns, as mentioned in the health technology assessment reports submitted to NICE (National Institute for Health and Care Excellence, 2012; National Institute for Health and Care Excellence, 2016). The critical concerns highlighted were the calculated risk of clinical events by considering the cohort’s baseline characteristics during randomisation and the assumption that the constant rate of hHF would overestimate the long-term efficacy of the medication. In addition, using the multi-state model over the two-state model would allow the clinical outcomes to be modelled using different stages of disease severity. The benefit of the treatment effect on disease progression can also be considered in the multi-state model.

The CEAs modelled from the Chinese medical and health system perspective reported that the ICER for empagliflozin + SoC compared to SoC alone was below the country’s 1xGDP *per capita* (Jiang et al., 2021; Lin et al., 2022; Tang and Sang, 2022). These studies simulated the CEM using the NYHA functional classification as the health state (Jiang et al., 2021; Lin et al., 2022; Tang and Sang, 2022). The problems associated with using the NYHA functional classification as a proxy for disease severity and progression were its non-reproducibility and subjectivity, as the NYHA assessment is not patient-centric and is subjected to interpersonal variation during evaluation by cardiologists (Raphael et al., 2007; Kosiborod et al., 2020). Together with the advantages mentioned in the methodology section, KCCQ is a more suitable tool to measure the progression of HF status.

The DSA results of the current study were robust to variation in the key model parameters and their plausible ranges. All the ICERs generated from the DSA were below the CET. The most impactful parameter that determined the cost-effectiveness of empagliflozin + SoC against SoC alone was the cost of empagliflozin. In the scenario analysis when the MoH acquisition costs of empagliflozin and other drugs were applied (i.e., lower than the average market price derived from the IQVIA database), the ICER reduced markedly from RM 20,400 per QALY in the base case to RM 6,627 per QALY. Thus, empagliflozin is highly cost-effective from the perspective of the MoH of Malaysia. The model also found that ICER was sensitive to CV death risk, and a slight change in this parameter would have a significant impact on the ICER (Jiang et al., 2021; Tang and Sang, 2022). The treatment effect of empagliflozin in preventing hHF was also the key driver that drove the cost-effectiveness of empagliflozin + SoC against SoC alone. The clinical efficacy of empagliflozin on the primary composite outcome was primarily driven by the reduction in the risk of hHF (Packer et al., 2020). Furthermore, hospitalisation severely impacts the HRQoL of HF patients because of the symptoms experienced by patients during the acute decompensated state, such as shortness of breath and lethargy (Albuquerque de Almeida et al., 2020). In addition, the risk of mortality has been shown to increase after each subsequent hHF (Lin et al., 2017). Thus, the clinical benefit of empagliflozin in averting hHF translates into more life years and QALYs gained, which significantly impact the ICER. These findings further explain the key parameters, such as the disutility associated with hHF, that drove the cost-effectiveness of empagliflozin + SoC relative to SoC alone. Apart from decreasing in a younger cohort, the ICERs also decreased when the time horizon increased. This indicates that the addition of empagliflozin to SoC has more pharmacoeconomic incentives, which result from the greater quantity of clinical events prevented and more life years and QALYs gained throughout HF patients’ lifespans. Following these scenarios, the ICERs were sensitive to the variations in the discount rate of costs and health outcomes. However, the findings from DSA and PSA confirmed that the ICERs were robust with respect to changes in key model parameters in the CEM.

In the scenario analysis, the ICER increased to RM 36,682 per QALY when the disease progression was modelled using the NYHA functional classes (RM 20,400 per QALY in the base-case scenario using KCCQ-CSS quartiles). The marked difference between the ICER for both models was primarily contributed by the distribution of patients based on the NYHA class relative to those in the KCCQ-CSS quartiles. In the NYHA model, about three-quarters of the patients entered the model with NYHA class II, and the remaining patients were split between NYHA class III and class IV (Supplementary Appendix A Table A1). In comparison, the distribution of patients was evenly spread across different degrees of disease severity when the disease progression was captured by the KCCQ-CSS quartiles. In addition, the monthly probability of transitioning out from NYHA class II starts low and decreases over time (Supplementary Appendix C Table C1), resulting in most patients remaining in the NYHA class II until the end of the simulation. Thus, fewer patients benefited from the treatment effect of empagliflozin + SoC in the NYHA class model, as captured in the risk equations for the clinical events (i.e., all-cause death, CV death, and hHF) and the improvement in health states. This finding is further confirmed by the fact that the life years and QALYs gained in the NYHA model were primarily contributed by patients in NYHA class II.

Despite clinical trials demonstrating the efficacies of SGLT2i in improving clinical outcomes in HFrEF patients (McMurray et al., 2019; Packer et al., 2020) and international guidelines advocating SGLT2i as part of guideline-directed medical therapy (GDMT) for HFrEF patients (McDonagh et al., 2021; Heidenreich et al., 2022), the SGLT2i prescribing rates reported in multiple observational studies are still low (Canonico et al., 2022; Chakrala et al., 2023; Okoroike et al., 2023). One potential barrier that prevents prescribing GDMT, including SGLT2i, is the medication acquisition costs (Canonico et al., 2022). Adding empagliflozin to SoC resulted in higher monthly medication costs and a longer life expectancy than SoC monotherapy, which increased lifetime medication and disease management costs even further. These additional costs, however, were partially offset by cost savings from avoiding hHF. The trade-offs between additional benefits gained and higher lifetime costs captured by ICER were well below the CET, indicating that empagliflozin + SoC was more cost-effective than SoC monotherapy. Adopting a cost-effective intervention, such as empagliflozin in this study, can improve the overall health of a population. Hence, the finding from this study confirms the economic effectiveness of empagliflozin in treating HF*r*EF and supports the decision to enlist the medication in the National Medicines Formulary. Consequently, this will improve access to the cost-effective medication and increase its prescribing rate.

The primary strength of the current study is that it is based on the EMPEROR-Reduced trial, wherein the background HF treatments in the comparator groups were reflective of the most recent HF treatment guidelines and closely resembled the suggested treatment regimen of HF*r*EF in Malaysia. This may increase the relevance of the results of this analysis. Secondly, the disease progression was captured by the patients’ self-reported KCCQ-CSS outcomes, which are both sensitive and reproducible in detecting changes in HF health status (Green et al., 2000; Spertus et al., 2005; Joseph et al., 2013) and a crucial secondary outcome in the EMPEROR-Reduced trial (Packer et al., 2020). This allows the granularity of the trial data to be captured. The risk of hHF can then be modelled based on the KCCQ-CSS health states.

The results of the analysis should be interpreted with its several limitations in mind. First, the long-term treatment effects of empagliflozin + SoC and SoC alone were extrapolated from the median follow-up duration of the EMPEROR-Reduced trial, resulting in uncertainty in the long-term estimates. Nevertheless, this is a limitation inherent to any CEM. The results of the sensitivity analyses suggest that the choice of parametric distribution for important clinical outcomes did not significantly influence the ICER. Therefore, it is unlikely that the uncertainties would change the conclusion of the study. Second, the treatment effects of empagliflozin on clinical events were estimated from the ITT population of the EMPEROR-Reduced trial. The uptake of medications could be different in the Malaysian clinical settings, especially the utilisation of ARNi (19.5% in the trial vs 6% in the Malaysia Heart Failure Registry (Ghazi et al., 2021)). Even so, the sub-group analysis of the EMPEROR-Reduced trial showed that the treatment effects of empagliflozin on the primary composite outcome (hHF or CV death) were consistent and independent of the baseline use of ARNi. Third, the CEM did not include diabetic ketoacidosis as one of the rare and recognised adverse effects of SGLT2i medications. This exclusion was supported by the fact that diabetic ketoacidosis was not observed in the EMPEROR-Reduced trial. Additionally, there was no imbalance in the incidence rates between the two treatment groups in the EMPA-REG OUTCOME trial involving T2DM patients with established CV diseases (Zinman et al., 2015). Fourth, the majority of the ITT population in the EMPEROR-Reduced trial was of Caucasian ethnicity, with Asian patients comprising approximately 18% of the total population. Moreover, the subgroup analysis showed that the risk reduction of the primary composite outcome was greater in the Asian population. Thus, the ICER values generated in the current study may represent a conservative estimate. Finally, we could not obtain data regarding the health utility of each health state in the Malaysian population, which may have potentially introduced some bias to the CEM outcomes. However, the ICERs were well below the CET even when the lower estimates of utility and disutility values were used in the one-way sensitivity analyses. In addition, the PSA was simulated using a wide range of health utilities to account for this difference. This further confirms the robustness of this finding as nearly all iterations were below the CET.

## 5 Conclusion

In conclusion, the CEM provided objective evidence that the addition of empagliflozin to SoC compared to SoC alone for the treatment of HF*r*EF was associated with improved clinical outcomes and HRQoL of patients with HF*r*EF at a reasonable upfront cost to pay. In the current study, empagliflozin + SoC was cost-effective from the perspective of the MoH of Malaysia. Considering the increasing prevalence of HF and especially the HFrEF population, cost-effective treatments such as empagliflozin could be important to the healthcare system.

## Data Availability

The original contributions presented in the study are included in the article/Supplementary Material, further inquiries can be directed to the corresponding author.
