# Supplementary material for: Cost-effectiveness of adding empagliflozin to the standard of care for patients with heart failure with reduced ejection fraction from the perspective of healthcare system in Malaysia
Source: Front Pharmacol. 2023 Jun 5;14:1195124. doi: 10.3389/fphar.2023.1195124 (PMC10277687; doi:10.3389/fphar.2023.1195124)
Supplement: Supplementary file 1 [file DataSheet1.docx]

Supplementary Material

Cost-effectiveness of Adding Empagliflozin to the Standard of Care for Patients with Heart Failure with Reduced Ejection Fraction from the Perspective of Healthcare System in Malaysia

Siew Chin Ong^1†*^, Joo Zheng Low^1,2†^, Stephan Linden^3^

^1^ Discipline of Social and Administrative Pharmacy, School of Pharmaceutical Sciences, Universiti Sains Malaysia, Malaysia

^2^ Hospital Sultan Ismail Petra, Ministry of Health, Malaysia

^3^ Boehringer Ingelheim International GmbH, Binger Str. 173, 55216, Ingelheim am Rhein, Germany. (Current Affiliation: CSL Behring, S.A. Tarragona 157, 18 floor, 08014 Barcelona, Spain)

*** Correspondence:**Siew Chin Ong
[siewchinong@usm.my](mailto:siewchinong@usm.my); [oschin99@yahoo.com](mailto:oschin99@yahoo.com)

**Appendix A. Base Model Description and Parameters Inputs**

Table A1. Mean cohort characteristics of ITT population at cost-effectiveness model entry

| **Baseline characteristic** | **ITT population** | **Standard error** |
| --- | --- | --- |
| **Demographics** | | |
| Age (years) | 60.0 ^a^ | 0.26 |
| Age (≥65 years) | 62.1% | 0.01 |
| Sex: Male | 76.1% | 0.01 |
| **Region** | | |
| Asia | 13.2% | 0.01 |
| Europe | 36.3% | 0.01 |
| Latin America | 34.5% | 0.01 |
| North America | 11.4% | 0.01 |
| Other | 4.6% | 0.00 |
| **KCCQ-CSS** |  |  |
| KCCQ-CSS 0 – 54 (Quartile 1) | 24.3% | 0.01 |
| KCCQ-CSS: 55 – 74 (Quartile 2) | 25.1% | 0.01 |
| KCCQ-CSS: 75 – 89 (Quartile 3) | 27.2% | 0.01 |
| KCCQ-CSS: 90 – 100 (Quartile 4) | 23.4% | 0.01 |
| **Baseline NYHA class** | | |
| NYHA II | 75.1% | 0.01 |
| NYHA III | 24.4% | 0.01 |
| NYHA IV | 0.5% | 0.00 |
| **Treatment used at baseline** | | |
| Angiotensin-converting enzyme inhibitors | 45.4% | 0.01 |
| Angiotensin receptor blockers | 24.3% | 0.01 |
| Angiotensin-receptor neprilysin inhibitor | 19.5% | 0.01 |
| Mineralocorticoid receptor antagonists | 71.3% | 0.01 |
| Beta-blockers | 94.7% | 0.00 |
| Ivabradine | 7.0% | 0.00 |
| **Medical history** | | |
| Ischaemic cause of heart failure | 51.7% | 0.01 |

CSS: clinical summary score; ITT: intent to treat; KCCQ: Kansas City Cardiomyopathy Questionnaire; NYHA: New York Heart Association

^a^ The mean age of the cohort was derived from the Malaysia Heart Failure registry (MyHF) [1].

Table A2. KCCQ-CSS monthly transition probabilities matrix

| **KCCQ-CSS Transitions [From, To]** | **Empagliflozin + SoC** | | | **SoC** | | |
| --- | --- | --- | --- | --- | --- | --- |
|  | **Months 1–3** | **Months 4–8** | **Months 9+** | **Months 1–3** | **Months 4–8** | **Months 9+** |
| KCCQ [1,1] | 0.796 | 0.910 | 0.918 | 0.834 | 0.903 | 0.929 |
| KCCQ [1,2] | 0.155 | 0.077 | 0.065 | 0.133 | 0.082 | 0.056 |
| KCCQ [1,3] | 0.025 | 0.005 | 0.013 | 0.014 | 0.009 | 0.013 |
| KCCQ [1,4] | 0.023 | 0.008 | 0.004 | 0.018 | 0.005 | 0.002 |
| KCCQ [2,1] | 0.066 | 0.068 | 0.051 | 0.069 | 0.058 | 0.051 |
| KCCQ [2,2] | 0.708 | 0.840 | 0.881 | 0.720 | 0.849 | 0.867 |
| KCCQ [2,3] | 0.188 | 0.083 | 0.061 | 0.203 | 0.079 | 0.076 |
| KCCQ [2,4] | 0.038 | 0.009 | 0.007 | 0.008 | 0.013 | 0.006 |
| KCCQ [3,1] | 0.004 | 0.005 | 0.004 | 0.013 | 0.011 | 0.013 |
| KCCQ [3,2] | 0.082 | 0.070 | 0.054 | 0.112 | 0.058 | 0.054 |
| KCCQ [3,3] | 0.771 | 0.848 | 0.868 | 0.743 | 0.859 | 0.871 |
| KCCQ [3,4] | 0.142 | 0.077 | 0.074 | 0.132 | 0.072 | 0.062 |
| KCCQ [4,1] | 0.006 | 0.004 | 0.003 | 0.006 | 0.004 | 0.000 |
| KCCQ [4,2] | 0.016 | 0.000 | 0.006 | 0.009 | 0.008 | 0.005 |
| KCCQ [4,3] | 0.074 | 0.063 | 0.044 | 0.096 | 0.058 | 0.049 |
| KCCQ [4,4] | 0.904 | 0.933 | 0.947 | 0.889 | 0.931 | 0.945 |

CSS: clinical summary score; KCCQ: Kansas City Cardiomyopathy Questionnaire; SoC: Standard of care

Table A3. Risk Equation for hospitalisation due to heart failure, all-cause death, CV death and empagliflozin discontinuation

| Predictors | Coefficients | Standard Error |
| --- | --- | --- |
| Hospitalisation due to heart failure |  |  |
| Intercept | -3.347 | 0.292 |
| Empagliflozin 10 mg (Ref.: Placebo) | -0.325 | 0.300 |
| KCCQ-CSS | | |
| KCCQ-CSS: 55-74 (Quartile 2) * | -0.450 | 0.279 |
| KCCQ-CSS: 75-89 (Quartile 3) * | -0.938 | 0.292 |
| KCCQ-CSS: 90-100 (Quartile 4) * | -1.352 | 0.341 |
| All-cause death |  |  |
| Distribution | Weibull |  |
| Distribution parameter (Shape) | 0.185 | 0.198 |
| Distribution parameter (Scale) | -8.695 | 0.288 |
| Empagliflozin 10 mg (Ref.: Placebo) | -0.044 | 0.275 |
| KCCQ-CSS |  |  |
| KCCQ-CSS: 55-74 (Quartile 2) * | -0.609 | 0.294 |
| KCCQ-CSS: 75-89 (Quartile 3) * | -1.161 | 0.322 |
| KCCQ-CSS: 90-100 (Quartile 4) * | -1.299 | 0.326 |
| Cardiovascular death |  |  |
| Distribution | Weibull |  |
| Distribution parameter (Shape) | 0.149 | 0.212 |
| Distribution parameter (Scale) | -8.667 | 0.307 |
| Empagliflozin 10 mg (Ref.: Placebo) | -0.059 | 0.295 |
| KCCQ-CSS |  |  |
| KCCQ-CSS: 55-74 (Quartile 2) * | -0.675 | 0.318 |
| KCCQ-CSS: 75-89 (Quartile 3) * | -1.183 | 0.344 |
| KCCQ-CSS: 90-100 (Quartile 4) * | -1.362 | 0.352 |
| Empagliflozin discontinuation |  |  |
| Distribution | Exponential |  |
| Intercept | 0.001 | 0.283 |
| Empagliflozin 10 mg (Ref.: Placebo) | -0.091 | 0.265 |
| KCCQ-CSS |  |  |
| KCCQ-CSS: 55-74 (Quartile 2) * | -0.519 | 0.282 |
| KCCQ-CSS: 75-89 (Quartile 3) * | -0.680 | 0.281 |
| KCCQ-CSS: 90-100 (Quartile 4) * | -1.099 | 0.304 |

CSS: clinical summary score; CV: cardiovascular; KCCQ: Kansas City Cardiomyopathy Questionnaire

* vs. KCCQ-CSS: 0 – 54 (Quartile 1)

Correlation between parameters of the risk equation were obtained from the variance-covariance matrices generated from the original regression equations.

Table A4. Rates of adverse events

| Event | Rate per 1,000 Patient Years in EMPEROR-Reduced Trial | |
| --- | --- | --- |
|  | **Empagliflozin + Standard of Care** | **Standard of Care** |
| Urinary tract infection | 41.3 | 37.6 |
| Genital mycotic infection | 13.8 | 5.3 |
| Acute renal failure | 81.3 | 90.2 |
| Hepatic injury | 34.3 | 38.3 |
| Volume depletion | 92.6 | 87.6 |
| Hypotension | 82.2 | 76.9 |
| Hypoglycaemic event | 12.0 | 12.5 |
| Bone fracture | 20.1 | 18.9 |

Table A5. Weighted average monthly cost for each pharmacological class

| **Pharmacological classes** | **Active ingredient** | **Share within-class (%) *** | **Weighted average monthly cost by active**  **ingredient (RM)** | **Weighted average monthly cost (RM)** |
| --- | --- | --- | --- | --- |
| ACEi | Captopril | 0.1 | 0.14 | 14.66 |
|  | Enalapril | 0.7 | 0.26 |  |
|  | Ramipril | 1.0 | 0.38 |  |
|  | Perindopril | 98.2 | 13.89 |  |
| ARB | Losartan | 65.0 | 70.87 | 95.79 |
|  | Valsartan | 35.0 | 24.92 |  |
| ARNi | Sacubitril/Valsartan | 100.0 | 386.88 | 386.88 |
| Beta-blockers | Bisoprolol | 86.8 | 37.73 | 43.44 |
|  | Carvedilol | 13.2 | 5.71 |  |
| MRA | Spironolactone | 100.0 | 13.93 | 13.63 |
| HCN channel blocker | Ivabradine | 100.0 | 187.36 | 187.36 |
| Diuretics | Furosemide | 95.0 | 2.89 | 6.64 |
|  | Bumetanide | 5 | 3.74 |  |

ACEi: angiotensin-converting enzyme inhibitor; ARB: angiotensin receptor blocker; ARNi: angiotensin receptor-neprilysin inhibitor; HCN: hyperpolarization-activated cyclic nucleotide-gated; MRA: mineralocorticoid antagonist; RM: Ringgit Malaysia

* Percentage of the utilisation of each active ingredient was obtained from an in-house local study [2].

Table A6. Weighted average cost of SoC and SoC + empagliflozin

| Pharmacological classes | ITT usage (%) | Monthly weighted average cost (RM) |
| --- | --- | --- |
| ACEi | 45.4 | 6.66 |
| ARB | 24.3 | 23.28 |
| ARNi | 19.5 | 75.44 |
| Beta-blockers | 94.7 | 41.14 |
| MRA | 71.3 | 9.93 |
| HCN channel blocker (Ivabradine) | 7.0 | 13.12 |
| Diuretics | 84.5 | 5.61 |
| Total (SoC) |  | 175.17 |
|  |  |  |
| Empagliflozin |  | 110.66 |
| SoC + Empagliflozin |  | 285.83 |

ACEi: angiotensin-converting enzyme inhibitor; ARB: angiotensin receptor blocker; ARNi: angiotensin receptor-neprilysin inhibitor; HCN: hyperpolarization-activated cyclic nucleotide-gated; ITT, intent to treat; MRA: mineralocorticoid antagonist; RM: Ringgit Malaysia; SoC: standard of care.

Table A7. Costs of hHF, CV death and disease management

| Event | Cost per event (RM) | Source |
| --- | --- | --- |
| hHF | 5,247 | [2] |
| CV Death ^ | 2,536 | [3] |
| Non-CV Death | 0 | Assumption |
| Disease management | Weighted monthly cost (RM) | Source |
| KCCQ-CSS Quartiles 1 – 4 | 28.28 ^#^ | [2] |

CSS: clinical summary score; CV: cardiovascular; hHF: hospitalisation due to heart failure; KCCQ: Kansas City Cardiomyopathy Questionnaire; RM: Ringgit Malaysia

^#^ Monthly outpatient visit frequency for all KCCQ-CSS quartiles were 0.2941 and monthly outpatient visit cost was RM 96.17. Thus, weighted monthly cost was 0.2941*RM 96.17 = RM 28.28

^ Detail derivation of CV death cost was described in Appendix B. The weighted cost was based on gender and number of fatal events.

Table A8: Costs of managing adverse events

| Adverse events | Outpatient Visit Cost, RM (Utilization %) | Inpatient Visit Cost, RM (Utilization %) | Weighted Average Cost (RM) |
| --- | --- | --- | --- |
| Urinary tract infection | 133 (99) | 3,863 (1) | 170 |
| Genital mycotic infection | 270 (99) | 5,174 (1) | 319 |
| Acute renal failure | 400 (20) | 3,859 (80) | 3,168 |
| Hepatic injury | 440 (20) | 3,906 (80) | 3,213 |
| Volume depletion | 400 (80) | 3,303 (20) | 981 |
| Hypotension | 320 (80) | 5,100 (20) | 1,276 |
| Hypoglycaemic event | 120 (90) | 5,815 (10) | 690 |
| Bone fracture | 6,395 (0) | 3,504 (100) | 3,504 |

RM: Ringgit Malaysia

Table A9. Utility and disutility inputs

| Parameter | Mean | SE | Duration | Source |
| --- | --- | --- | --- | --- |
| KCCQ-CSS Quartile 1 | 0.6043 | 0.0040 | Permanent | Based on EMPEROR-Reduced trial data analyses |
| KCCQ-CSS Quartile 2 | 0.7211 | 0.0030 | Permanent |  |
| KCCQ-CSS Quartile 3 | 0.7942 | 0.0030 | Permanent |  |
| KCCQ-CSS Quartile 4 | 0.8581 | 0.0030 | Permanent |  |
| Clinical event disutility |  |  |  |  |
| hHF | -0.2459 | 0.062 | 1 month | Based on EMPEROR-Reduced trial data analyses |
| Adverse event disutilities |  |  |  |  |
| Urinary tract infection | –0.025 | 0.027 | 1 month | [4] |
| Genital mycotic infection | –0.038 | 0.077 | 1 month | [4] |
| Acute renal failure | –0.038 | 0.075 | 1 month | [4] |
| Hepatic injury | –0.016 | 0.020 | 1 month | Based on EMPEROR-Reduced trial data analyses |
| Volume depletion | –0.018 | 0.015 | 1 month | Based on EMPEROR-Reduced trial data analyses |
| Hypotension | –0.025 | 0.000 | 1 month | [5] |
| Hypoglycaemic event | –0.005 | 0.010 | 1 month | [6] |
| Bone fracture | –0.165 | 0.037 | 1 month | Based on EMPEROR-Reduced trial data analyses |

CSS: clinical summary score; hHF: hospitalisation due to heart failure; KCCQ: Kansas City Cardiomyopathy Questionnaire; SE: standard error.

Table A10. Parameters and distributions for probabilistic sensitivity analysis

| **Parameter** | **Probabilistic distribution** |
| --- | --- |
| Risk equations for hHF, all-cause death, CV death, and treatment discontinuation ^a^ | Correlated draws from multivariate normal distributions from Cholesky decomposition of covariance matrices |
| KCCQ-CSS transition probabilities ^b^ | Dirichlet |
| Adverse events rates ^c^ | Gamma |
| Utility by health state ^d^ | Beta |
| Utility decrements ^d^ | Beta |
| Clinical event management costs ^e^ | Gamma |
| Disease management costs ^f^ | Gamma |
| Medication acquisition costs ^g^ | Gamma |

CSS: clinical summary score; CV: cardiovascular; hHF: hospitalisation duet to heart failure; KCCQ: Kansas City Cardiomyopathy Questionnaire

^a^ Risk equations are shown in Table A3

^b^ Transition probabilities are shown in Table A2

^c^ Adverse event rates are shown in Table A4

^d^ Utility values are shown in Table A9

^e^ Costs are shown in Table A7

^f^ Costs are shown in Table A7

^g^ Costs are shown in Table A6

**Appendix B: Derivation of Cardiovascular Death Cost**

Table B1. Cost of fatal events categorised by gender

| **Fatal Events** | **Cost of fatal event in 2008 (RM)** | | **Gender weighted cost (RM)** |
| --- | --- | --- | --- |
|  | **Male (75%)** | **Female (25%)** |  |
| Major coronary | 601 | 619 | 605 |
| Major cerebrovascular | 3,556 | 3,730 | 3,599 |
| Heart failure | 831 | 858 | 838 |

RM: Ringgit Malaysia

Cost of fatal event in 2008 was obtained from Clarke et al for Malaysia setting [3].

Table B2. Cost of cardiovascular death

| **Fatal Events** | **Number of cases in 2020** | **Gender weighted cost** | | **Average weighted CV death cost (RM)** |
| --- | --- | --- | --- | --- |
|  |  | **Year 2008** | **Year 2021*** |  |
| Major coronary | 2,668 | 605 | 784 | 2,536 |
| Major cerebrovascular | 2,822 | 3,599 | 4,661 |  |
| Heart failure | 911 | 838 | 1,085 |  |

CV: cardiovascular; RM: Ringgit Malaysia

* Cost was inflated using consumer price index health domain

**Appendix C: NYHA Model and Parameters**


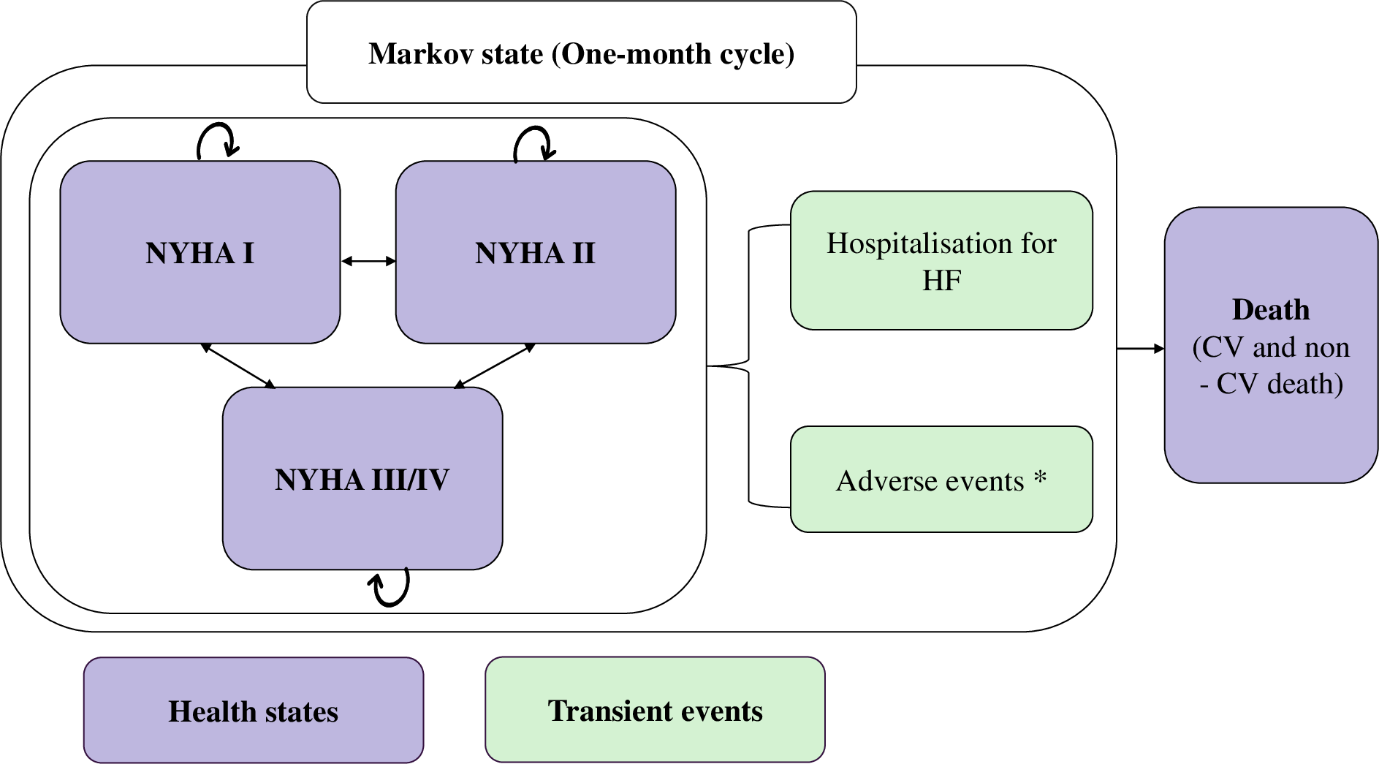


CV: cardiovascular; hHF: hospitalisation due to heart failure; NYHA: New York Heart Association

Adverse events: urinary tract infection, genital mycotic infection, acute renal injury, hepatic injury, hypotension, hypoglycaemic event, and bone fracture.

Figure E1. Cost Effectiveness Model Structure based on NYHA Functional Classes

Table C1. NYHA monthly transition probabilities matrix

| NYHA Transitions [From, To] | Empagliflozin + Standard of Care | | | Standard of Care | | |
| --- | --- | --- | --- | --- | --- | --- |
|  | Months 1–3 | Months 4–8 | Months 9+ | Months 1–3 | Months 4–8 | Months 9+ |
| NYHA [I, III/IV] | 0.005 | 0.003 | 0.002 | 0.006 | 0.000 | 0.004 |
| NYHA [I, II] | 0.125 | 0.060 | 0.044 | 0.205 | 0.086 | 0.064 |
| NYHA [I, I] | 0.870 | 0.937 | 0.954 | 0.790 | 0.914 | 0.932 |
| NYHA [II, III/IV] | 0.022 | 0.011 | 0.011 | 0.034 | 0.016 | 0.011 |
| NYHA [II, II] | 0.928 | 0.972 | 0.977 | 0.923 | 0.971 | 0.978 |
| NYHA [II, I] | 0.050 | 0.017 | 0.012 | 0.043 | 0.013 | 0.011 |
| NYHA [III/IV, III/IV] | 0.726 | 0.948 | 0.941 | 0.755 | 0.926 | 0.944 |
| NYHA [III/IV, II] | 0.265 | 0.052 | 0.058 | 0.232 | 0.071 | 0.054 |
| NYHA [III/IV, I] | 0.009 | 0.000 | 0.001 | 0.012 | 0.004 | 0.002 |

NYHA: New York Heart Association

Table C2. Risk equations for hospitalisation due to heart failure, all-cause death, CV death and empagliflozin discontinuation (NYHA model)

| Predictors | Coefficients | Standard Error |
| --- | --- | --- |
| Hospitalisation due to heart failure |  |  |
| Intercept | -3.155 | 0.300 |
| Empagliflozin 10 mg (Ref.: Placebo) | -0.316 | 0.294 |
| NYHA class (Ref.: Class III–IV) | | |
| Class II | -1.126 | 0.237 |
| Class I | -1.484 | 0.430 |
| All-cause death |  |  |
| Distribution | Weibull |  |
| Distribution parameter 1 (Shape) | 0.194 | 0.197 |
| Intercept | -8.691 | 0.290 |
| Empagliflozin 10 mg (Ref.: Placebo) | -0.036 | 0.278 |
| NYHA class (Ref.: Class III–IV) |  |  |
| Class II | -1.025 | 0.239 |
| Class I | -1.560 | 0.452 |
| Cardiovascular death |  |  |
| Distribution | Weibull |  |
| Distribution parameter 1 (Shape) | 0.157 | 0.212 |
| Intercept | -8.517 | 0.310 |
| Empagliflozin 10 mg (Ref.: Placebo) | -0.043 | 0.298 |
| NYHA class (Ref.: Class III–IV) |  |  |
| Class II | -1.038 | 0.257 |
| Class I | -1.502 | 0.479 |
| Empagliflozin discontinuation |  |  |
| Distribution | Exponential |  |
| Distribution parameter 1 | 0.000 | 0.191 |
| Intercept | 0.001 | 0.283 |
| Empagliflozin 10 mg (Ref.: Placebo) | -0.083 | 0.265 |
| NYHA class (Ref.: Class III–IV) |  |  |
| Class II | -0.595 | 0.282 |
| Class I | -1.334 | 0.281 |

CV: cardiovascular; NYHA: New York Heart Association

Table C3. Utility and disutility inputs

| Parameter | Mean Utility | SE | Source |
| --- | --- | --- | --- |
| NYHA III / IV | 0.6981 | 0.0050 | Based on EMPEROR-Reduced trial data analyses |
| NYHA II | 0.7739 | 0.0026 |  |
| NYHA I | 0.8080 | 0.0054 |  |
| Clinical event disutility |  |  |  |
| hHF | -0.3366 | 0.062 | Based on EMPEROR-Reduced trial data analyses |

NYHA: New York Heart Association; hHF: hospitalisation due to heart failure; SE: standard error

Table C4. Disease management cost based on NYHA functional classes

| NYHA class | Monthly visit frequency | Monthly cost (RM) | Weighted monthly cost (RM) | |
| --- | --- | --- | --- | --- |
| I | 0.3187 | 96.83 | | 30.86 |
| II | 0.2630 | 95.25 | | 25.05 |
| III/IV | 0.3243 | 112.92 | | 36.62 |

NYHA: New York Heart Association; RM: Ringgit Malaysia

**Appendix D: Additional results for base case analysis**

Table D1. Deterministic sensitivity analysis inputs and results

| Parameters | Base-Case Input | Sensitivity Analysis Input | ICER (RM/QALY) |
| --- | --- | --- | --- |
| Base case scenario | - | - | 20,400 |
| Clinical Inputs |  |  |  |
| CV & All-cause death: Distribution | Weibull | Exponential | 21,027 |
| CV mortality: Treatment effect | -0.0589 | 0 | 24,949 |
|  |  | -0.2877 | 14,346 |
| All-cause death: Adjust with Malaysia lifetable? | Yes | No | 18,024 |
| All-cause death: Treatment effect | -0.0444 | 0 | 21,182 |
|  |  | -0.2614 | 19,763 |
| hHF: Treatment effect | -0.3246 | -0.1625 | 25,684 |
|  |  | -0.5447 | 15,722 |
| Discontinuation: Distribution | Exponential | Weibull | 20,644 |
| Include discontinuation? | Yes | No | 21,260 |
| Costs and Resource Use |  |  |  |
| Cost of hHF (RM) | 5,247 | 4,010 | 21,655 |
|  |  | 6,648 | 18,978 |
| Cost of CV death (RM) | 2,536 | 2,029 | 20,446 |
|  |  | 3,043 | 20,354 |
| Monthly Cost of Disease Monitoring: KCCQ-CSS Quartile (RM) | 28.28 | 18.3 | 20,447 |
|  |  | 40.4 | 20,342 |
| Monthly Cost of Disease Monitoring: KCCQ-CSS Quartile 2 (RM) | 28.28 | 18.3 | 20,379 |
|  |  | 40.4 | 27,580 |
| Monthly Cost of Disease Monitoring: KCCQ-CSS Quartile 3 (RM) | 28.28 | 18.3 | 20,416 |
|  |  | 40.4 | 20,380 |
| Monthly Cost of Disease Monitoring: KCCQ-CSS Quartile 4 (RM) | 28.28 | 18.3 | 20,264 |
|  |  | 40.4 | 20,463 |
| Cost of AE management (RM) | Multiple values | -20% | 20,375 |
|  |  | +20% | 20,425 |
| Empagliflozin cost per pack (RM) | 110.66 | 77.5 | 13,220 |
|  |  | 143.9 | 27,580 |
| Utilities |  |  |  |
| Utility: KCCQ-CSS Quartile 1 | 0.6043 | 0.5964 | 20,336 |
|  |  | 0.6121 | 20,463 |
| Utility: KCCQ-CSS Quartile 2 | 0.7211 | 0.7152 | 20,421 |
|  |  | 0.7270 | 20,379 |
| Utility: KCCQ-CSS Quartile 3 | 0.7942 | 0.7883 | 20,384 |
|  |  | 0.8001 | 20,416 |
| Utility: KCCQ-CSS Quartile 4 | 0.8581 | 0.8522 | 20,538 |
|  |  | 0.8640 | 20,266 |
| Disutility: hHF | -0.2459 | -0.1357 | 22,969 |
|  |  | -0.3763 | 18,015 |
| Disutility: AEs | Multiple values | Lower 95% CI | 20,377 |
|  |  | Upper 95% CI | 20,445 |
| Settings |  |  |  |
| Discount rate: cost | 3% | 0 | 22,860 |
|  |  | 5% | 19,045 |
| Discount rate: health | 3% | 0 | 17,429 |
|  |  | 5% | 22,430 |

AEs: adverse events; CV: cardiovascular; CI: confidence interval; hHF: hospitalisation for heart failure; ICER: Incremental cost-effectiveness ratio; QALY: quality-adjusted life-year; KCCQ-CSS: Kansas City Cardiomyopathy Questionnaire Clinical Summary Score; RM: Ringgit Malaysia

Table D2. Summary of probabilistic sensitivity analysis results for empagliflozin + SoC vs. SoC

| **Outcome** | **Empagliflozin + SoC** | **SoC** | **Incremental** |
| --- | --- | --- | --- |
| Mean total cost (RM) | 24,347 | 21,314 | 3,033 |
| Mean total QALYs | 3.58 | 3.43 | 0.15 |
| ICER, Cost per QALY gained (RM) | 20,266 |  | |

QALY: quality-adjusted life-year; RM: Ringgit Malaysia; SoC: standard of care

**Appendix E. Results of Scenario Analysis using NYHA as health states**

Table E1. Base case results for the cost-effectiveness of adding empagliflozin to the standard of care (NYHA model)

| **Outcome** | **Empagliflozin + SoC** | **SoC** | **Incremental** |
| --- | --- | --- | --- |
| Total Cost (RM) | 23,970 | 20,575 | 3,395 |
| Total LYs | 4.71 | 4.65 | 0.05 |
| Total QALYs | 3.37 | 3.28 | 0.09 |
| ICER, Cost per LY gained (RM/LY) | 62,986 | | |
| ICER, Cost per QALY gained (RM/QALY) | 36,682 | | |

ICER: incremental cost-effectiveness ratio; LY: life years; NYHA: New York Heart Association; QALYs: quality-adjusted life years; RM: Ringgit Malaysia; SoC: standard of care;

Table E2. Summary of clinical and cost outcomes for the scenario using NYHA health states

| **Clinical Outcomes** | **Empagliflozin + SoC** | **SoC** | **Incremental** |
| --- | --- | --- | --- |
| **Event rates (per 100 patient-years)** | | | |
| HF hospitalisation | 16.30 | 19.64 | -3.33 |
| CV death | 11.03 | 11.29 | -0.26 |
| Non-CV death | 7.73 | 7.70 | 0.02 |
| **Adverse events** |  |  |  |
| Urinary tract infection | 3.99 | 3.76 | 0.23 |
| Genital mycotic infection | 1.07 | 0.53 | 0.54 |
| Acute renal failure | 8.46 | 9.02 | -0.56 |
| Hepatic injury | 3.58 | 3.83 | -0.25 |
| Volume depletion | 9.08 | 8.76 | 0.32 |
| Hypotension | 8.03 | 7.69 | 0.34 |
| Hypoglycaemic event | 1.22 | 1.25 | -0.03 |
| Bone fracture | 1.97 | 1.89 | 0.08 |
| **Time on treatment (undiscounted), LYs and QALYs (discounted) per patient** | | | |
| Time receiving empagliflozin (years) |  |  |  |
| **Total LYs** | **4.71** | **4.65** | **0.05** |
| NYHA I | 0.54 | 0.53 | 0.01 |
| NYHA II | 3.33 | 3.29 | 0.04 |
| NYHA III/IV | 0.83 | 0.83 | 0.01 |
| **Total QALYs** | **3.37** | **3.28** | **0.09** |
| NYHA I | 0.38 | 0.37 | 0.01 |
| NYHA II | 2.58 | 2.55 | 0.03 |
| NYHA III/IV | 0.67 | 0.67 | 0.01 |
| Loss due to hHF | -0.257 | -0.308 | 0.05 |
| Loss due to AEs | -0.005 | -0.005 | 0.00 |
| **Cost Outcomes** | **Empagliflozin + SoC** | **SoC** | **Incremental** |
| Cost outcomes (discounted), per patient | | | |
| **Drug acquisition cost (RM)** | **14,008** | **9,783** | **4,225** |
| **Clinical event management cost (RM)** | 5,300 | 6,116 | -816 |
| HF hospitalisation | 4,006 | 4,805 | -799 |
| CV death | 1,294 | 1,310 | -16 |
| **AE management cost (RM)** | **3,113** | **3,145** | **-32** |
| Urinary tract infection | 32 | 30 | 2 |
| Genital mycotic infection | 16 | 8 | 8 |
| Acute renal failure | 1,258 | 1,330 | -72 |
| Hepatic injury | 540 | 573 | -33 |
| Volume depletion | 420 | 400 | 20 |
| Hypotension | 483 | 457 | 26 |
| Hypoglycaemic event | 40 | 40 | -1 |
| Bone fracture | 325 | 308 | 17 |
| **Disease management cost (RM)** | **1,549** | **1,531** | **18** |
| NYHA I | 238 | 235 | 3 |
| NYHA II | 1,001 | 990 | 11 |
| NYHA III/IV | 309 | 306 | 3 |
| **Total cost (RM)** | **23,970** | **20,575** | **3,395** |

AE: adverse events; CV: cardiovascular; HF: heart failure; hHF: hospitalisation due to heart failure; LY: life year; NYHA: New York Heart Association; QALY: quality-adjusted life year; SoC: standard of care

**References**

1. Abidin HAZ, Kader MASA, Ross NT, Ramli AW, Ghazi AM, Hassan HHC*, et al.* Demographic characteristics differences across EF subgroups in the Malaysian heart failure (MyHF) registry. Int J Cardiol. 2021;**345**:5-6. doi.org/<https://doi.org/10.1016/j.ijcard.2021.10.040>

2. Ong SC, Low JZ, Yew WY, Yen CH, Muhamad AS, Liew HB*, et al.* EE141 Cost Analysis of Heart Failure Management in Malaysia: A Multi-Centered Retrospective Study. Value Health. 2022;**25**(7, Supplement):S361-S2. doi.org/<https://doi.org/10.1016/j.jval.2022.04.391>

3. Clarke PM, Glasziou P, Patel A, Chalmers J, Woodward M, Harrap SB*, et al.* Event rates, hospital utilization, and costs associated with major complications of diabetes: a multicountry comparative analysis. PLoS Med. 2010;**7**(2):e1000236. doi.org/10.1371/journal.pmed.1000236

4. Sullivan PW, Ghushchyan VH. EQ-5D Scores for Diabetes-Related Comorbidities. Value Health. 2016;**19**(8):1002-8. doi.org/10.1016/j.jval.2016.05.018

5. Sullivan PW, Ghushchyan V. Preference-Based EQ-5D index scores for chronic conditions in the United States. Med Decis Making. 2006;**26**(4):410-20. doi.org/10.1177/0272989X06290495

6. National Institute for Health and Care Excellence. Type 2 diabetes: newer agents for blood glucose control in type 2 diabetes. NICE short clinical guideline. 2011.
